# Factors associated with physical activity reduction in Swedish older adults during the first COVID-19 outbreak: a longitudinal population-based study

**DOI:** 10.1186/s11556-022-00287-z

**Published:** 2022-04-01

**Authors:** Linnea Sjöberg, Federico Triolo, Marguerita Saadeh, Serhiy Dekhtyar, Amaia Calderón-Larrañaga, Anna-Karin Welmer

**Affiliations:** 1 Aging Research Center, Department of Neurobiology, Care Sciences and Society, Karolinska Institutet and Stockholm University, Tomtebodavägen 18ASE-171 65, Solna, Stockholm, Sweden; 2 Stockholm Gerontology Research Center, Stockholm, Sweden; 3 Women´s Health and Allied Health Professionals Theme, Medical Unit Medical Psychology, Karolinska University Hospital, Stockholm, Sweden; 4 Division of Physiotherapy, Department of Neurobiology, Care Sciences and Society, Karolinska Institutet, Stockholm, Sweden

**Keywords:** COVID-19, Physical Activity, Risk factors, Population-based study, Older adults, Epidemiology

## Abstract

**Background:**

Physical activity (PA) decreased during the COVID-19 pandemic, especially among older adults, potentially leading to adverse consequences for their health. However, factors associated with reductions of PA during the pandemic have not been examined in a population-based sample of older adults. Thus, the aim of this study was to explore the association of pre-pandemic physical, mental, social and lifestyle factors with reductions in PA in older adults during the first wave of COVID-19, and whether the associations differed by age and sex.

**Methods:**

A population-based sample of 624 participants aged 65-99 years were identified from the Swedish National study on Aging and Care in Kungsholmen (SNAC-K) COVID19 Study. Information on pre-pandemic factors was collected through clinical examinations, interviews, and self-administered questionnaires in 2016-2019. Changes in light and intense PA during the first wave of the pandemic (May-September 2020) were self-reported. Data were analyzed using multiple logistic regression models, stratified by age (<70 vs. >80 years) and sex.

**Results:**

There was an association between pre-pandemic levels of higher depressive symptom burden (Odds Ratio (OR): 2.6, 95% Confidence Interval (CI): 1.1-6.4, <70 years), and impaired balance (OR: 1.7, 95% CI: 1.0-2.8, >80 years old) with reductions in light-intensity PA. Furthermore, the presence of musculoskeletal disease (OR: 1.8, 95% CI: 1.1-2.9, <70 years; OR: 2.3, 95% CI: 1.2-4.4, men), moderate/high levels of neuroticism (OR: 1.6, 95% CI: 1.0-2.6, <70 years; OR: 2.2, 95% CI: 1.3-3.5, women), and poor levels of social support (OR: 2.2, 95% CI: 1.2-4.3, >80 years) were related to reductions in higher-intensity PA. Those who were current smokers (OR: 0.3, 95% CI: 0.1-0.8, <70 years; OR: 0.2, 95% CI: 0.06-0.7, women), or had impaired balance (OR: 0.4, 95% CI: 0.2-0.8, >80 years) were less likely to reduce their levels of higher-intensity PA.

**Conclusions:**

For future pandemics or waves of COVID-19, development of strategies is warranted for older individuals with psychiatric- or physical illness/dysfunction, as well as those with poor social support to counteract reductions in physical activities.

**Supplementary Information:**

The online version contains supplementary material available at 10.1186/s11556-022-00287-z.

## Introduction

The pandemic caused by the novel coronavirus, SARS-CoV-2, has become an extraordinary public health emergency [1]. It has led to the implementation of strict measures to mitigate the quick spread of the infection and reduce mortality. Swedish authorities relied on high voluntary adherence to the recommendations by the Public Health Agency rather than forcing people to stay at home [2]. The recommendations included social distancing and avoiding physical contact even when having slight symptoms. Older adults were advised to follow stricter social isolation recommendations, as compared to younger individuals [3].

Although intended to reduce the morbidity and mortality linked to SARS-CoV-2 infection, such preventive measures may have led to indirect damage like worsening of lifestyle habits and reduced physical activity (PA). Results from an international online survey, which included 1047 individuals from Europe (21%), Africa (40%), Asia (36%), and others (3%), suggested that the social isolation promoted during the COVID-19 outbreak resulted in a 24% reduction of light-, moderate- or vigorous-intensity PA in adults aged 18-55+ years [4]. Similarly, results from an internet-based survey in a Brazilian sample of volunteers aged 30-48 years found that 36% had reduced their PA levels during the pandemic [5]. Moreover, pandemic-related reductions of PA have also been reported by 34% of older adults (mean age=78 years) who participated in the Finnish Geriatric Intervention Study to Prevent Cognitive Impairment and Disability (FINGER) [6], as well as by one third of Swedish older adults aged 68+ years in a study from our group [7]. This reduction may impose an important threat to the health of older adults given the well-known protection conferred by PA against chronic diseases and premature death [8, 9], and emphasizes the need to develop strategies to avoid such declines in the older population.

Characterizing the population that may have undergone reductions of PA during the pandemic is crucial for the development of strategies aimed at resuming pre-pandemic levels. Peréz, et al. investigated predictors of PA levels during the first wave of the pandemic in a sample of 98 Spanish frail older adults. They found that individuals with reduced or insufficient PA were more likely to have higher pre-pandemic levels of depressive symptoms, cognitive impairment/dementia, disability, comorbidity, and less social contacts than those who improved or maintained sufficient PA during the first COVID-19 outbreak [10]. Furthermore, the pandemic-related reduction of PA among older adults in the FINGER study was more evident among those who were living alone [6].

So far however, factors associated with a reduction of PA levels have not been examined in a population-based sample of older adults. Previous studies have shown that PA levels in the general population of older adults differ according to physical, mental, social and lifestyle factors [11, 12]. Thus, the aim of this study was to explore the association of pre-pandemic levels of physical, mental, social and lifestyle factors with PA reduction during the first pandemic outbreak in Swedish older adults. Since previous research shows that PA levels generally decrease with age and that older men are more likely than older women to participate in regular PA [13, 14], we further examined whether the associations differed by age and sex.

## Methods

### Study population

This study used data from the Swedish National Study on Aging and Care in Kungsholmen (SNAC-K) COVID19 Study (May-September 2020), and the data were linked to the sixth wave of follow-up (2016-2019) in SNAC-K [15]. SNAC-K is a longitudinal study that includes a random sample of older adults from 11 age cohorts (60, 66, 72, 78, 81, 84, 87, 90, 93, 96, and ≥99 years) who live in the Kungsholmen district of Stockholm. The eligible baseline sample included 4590 individuals [16]. Of these, 3363 participated (73.3 %) in the baseline examination in year 2001-2004 and have been followed up regularly every 6 years for those aged ≤72 years and every 3 years for those aged ≥78 years. Additional cohorts were added in 2007-2010 (81-year-olds, *N*=194), 2010-2013 (60-year-olds, *N*=677), and 2013-2016 (81-year-olds, *N*=195).

All participants eligible for the seventh SNAC-K wave (2019-2021) were invited to participate in a telephone interview between May and September 2020 (*n*=1442), when COVID-19 was declared a pandemic [17] (95% of the interviews were conducted in May and June). Individuals who had previous cognitive or physical difficulties, lived in nursing homes, or who had deceased since the sixth wave of follow-up were excluded from the SNAC-K COVID19 Study (*n*=103), leaving 1339 individuals. Out of these, 108 persons refused to participate or could not be contacted (response rate 91.9%).

Out of the 1231 individuals who participated in the SNAC-K COVID19 Study, 951 had also participated in wave 6 (2016-2019) (age cohorts 66, 81, 84, 87, 90, 93 and ≥96 years). Wave 6 will be referred to as the baseline examination of this study. For the purpose of this study, individuals having a Mini-Mental-State-Examination (MMSE) <24 [18] (*n*=18) and those who had a positive COVID-19 test (*n*=8) were excluded from the sample. We also excluded persons with missing data regarding the questions on COVID-19 testing (*n*=1), changes in PA levels during the pandemic (*n*=40), or in pre-pandemic factors, i.e., the Montgomery Åsberg Depression Scale (MADRS, *n*=17) [19], social network (*n*=87), physical functioning (*n*=82), lifestyle factors (*n*=29), or personality traits (*n*=45), leaving 624 individuals for the analyses.

Informed written consent was collected directly from each participant. The Regional Ethics Review Board in Stockholm has approved all phases of the SNAC-K study, as well as the SNAC-K COVID19 study (dnr: 2020-02497).

### Data collection

During the baseline examination, information was collected through clinical examinations, nurse interviews, self-administered questionnaires, and cognitive tests administered by physicians, nurses, and psychologists. Information on physical health and behavioral conditions during the COVID-19 pandemic were obtained by trained nurses through telephone interview, following standard protocols.

### Outcome

During the telephone interview, a nurse asked the participants whether they had changed their levels of light- or higher-intensity PA during the pandemic, as compared to their pre-pandemic levels (before March 2020). Light PA included walking, short bike rides, light gymnastics, and golfing. Higher-intensity PA included activities such as jogging, brisk long walks or bike rides, heavy gardening, intense gymnastics, long distance skating, skiing, or swimming. The response alternatives were “more”, “the same/no change”, or “less”. Since the focus of this study was to investigate PA reductions during the pandemic, and given that the proportion of those engaging in increased PA levels was the smallest group in the total sample (increases in light PA=21% and in intense PA=10%), PA was categorized as a reduction in PA (“less”) versus no reduction (“more or the same/no change”) in light or intense PA levels, respectively.

### Exposures

The exposures were assessed during the baseline examination in 2016-2019.

#### Social network

Both quantity and quality of social network were measured. For social connections (quantity), questions on marital status, cohabitation status, parenthood, friendships, social network size, and frequency of direct contacts with children, parents, neighbors, and friends were included. Social support (quality) was assessed by the following items: reported satisfaction with the above-mentioned contacts, perceived material and psychological support, sense of affinity with association members, relatives, and living area, and being part of a group of friends. The overall scores for each variable were transformed into z-scores [20], and categorized according to the median as poor (below median) versus good (median or above).

#### Chronic somatic and mental diseases

Disease diagnoses were coded based on the International Classification of Diseases Tenth Revision (ICD-10) [21], and further operationalized according to a methodology described previously [22]. For the purpose of this study, 23 chronic cardiovascular, musculoskeletal, and neuropsychiatric diseases were included given their major contribution to functional impairment and disability [23], and their tendency to cluster together into recognized multimorbidity patterns [24]. The specific conditions considered were: 1) cardiovascular disease, i.e., ischemic heart disease, heart failure, atrial fibrillation, cerebrovascular diseases, cardiac valve diseases, bradycardias or conduction diseases, peripheral vascular disease, and other cardiovascular diseases; 2) musculoskeletal disease, i.e., dorsopathies, inflammatory arthropathies, osteoarthritis and other degenerative joint diseases, osteoporosis, and other musculoskeletal and joint diseases; and 3) neuropsychiatric disease, i.e., dementia, neurotic or stress-related and somatoform diseases, migraine and facial pain syndromes, peripheral neuropathy, Parkinson or parkinsonism, epilepsy, schizophrenia and delusional diseases, multiple sclerosis, other psychiatric or behavioral diseases, and other neurological diseases. The presence of cardiovascular, musculoskeletal, or neuropsychiatric diseases were categorized as having any versus having no disease.

Global cognition was measured using the Mini-Mental State Examination (MMSE) and was classified as MMSE scores of 24-27 versus 28-30. The Montgomery-Åsberg Depression Rating Scale (MADRS) was used to assess depressive symptoms [19]. The 10-item MADRS has a maximum score of 60 and the cut-off for depressive symptoms was >6 [25].

#### Physical functioning

The chair stand test was performed by asking participants to raise up from a chair five times as fast as possible, without using their arms, and the results were expressed in seconds. Walking speed at a normal pace was assessed over 6 m or 2.44 m, depending on the participants’ ability and the location of the test, and presented in meters per seconds (m/s). Balance was assessed using the one-leg balance test where participants were asked to stand twice on each leg up to 60 seconds, with their eyes open. Impaired mobility was defined as a walking speed <0.8 meter/seconds [26], impaired muscle strength as a chair stand time ≥17 seconds [27], and impaired balance as a single leg standing time <5 seconds [28]. Participants who were unable to perform a test due to severe physical limitations were categorized as having impaired function in that test [29].

#### Lifestyle factors

Smoking was categorized as being a smoker versus non-smoker. Alcohol consumption was obtained according to the frequency and amount consumed and was classified as none/occasional, light to moderate (1-14 drinks per week for men and 1-7 drinks per week for women), or heavy (>14 drinks per week for men or >7 drinks per week for women) [30]. Body Mass Index (BMI) was classified as underweight (<20 kg/m^2^), normal weight (≥20-<25 kg/m^2^), or overweight/obesity (≥25 kg/m^2^) [31]. For the analyses, alcohol consumption was dichotomized as no/occasional or heavy versus light to moderate, and BMI as underweight or overweight/obesity versus normal weight since these subcategories were similarly associated with reductions in PA. This dichotomization is further supported by the literature since studies have found J-shaped associations of alcohol consumption and BMI with adverse health outcomes in older age [32–34].

#### Personality traits

The personality traits of neuroticism, extraversion, and openness to experience were obtained using a 36 item from short version of the NEO Five-Factor Inventory (NEO-FFI). The scores were transferred into z-scores and categorized as follows: neuroticism as moderate or high versus low, and extraversion or openness to experience as low versus moderate or high [35].

### Covariates

#### Demographic factors

Demographic factors included age, sex, and education. Education refers to the highest level of education achieved and was classified as elementary, high school, or university. For the analyses, education was dichotomized as having high school or less versus more than high school education since elementary and high school education were similarly associated with reductions in PA.

#### Physical activity at baseline

Light or intense PA in the 12 months prior to baseline was assessed by self-report. Light and intense PA were equally defined in the pre-pandemic baseline interview and the COVID-19 telephone interview. The answers for light and intense PA were combined and categorized as follows: insufficient levels of PA (less than weekly engagement in light and intense exercise) versus sufficient levels (engagement in light or/and intense exercise several days per week or more) [36].

### Statistical analyses

Differences in the proportions of the baseline characteristics by age and sex were evaluated using Chi-Square tests for categorical data. For continuous data, Mann-Whitney U tests were used because of the skewed distributions. Multiple logistic regression models were used to examine the associations between pre-pandemic levels of physical, mental, social, and lifestyle-related factors with light and intense PA reduction during the pandemic. First, each potential risk factor was entered separately, adjusting for age, sex, and education. Second, analyses were performed to examine the multiplicative interaction between each potential risk factor with age and sex. Third, all risk factors associated with a reduction in PA with P-values <0.1 were entered in multiple regression models using a backward selection procedure. There was no multicollinearity between the exposure variables as the variance inflation factors (VIFs) were <4. For all statistical tests, *P*-values ≤0.05 were considered statistically significant, and all statistical tests were two-tailed. The statistical analyses were performed using SPSS 26.

### Sensitivity analyses

To further explore the independent effect of the pre-pandemic risk factors on PA reduction during the pandemic, baseline PA were additionally adjusted for in the fully adjusted models.

## Results

Baseline characteristics and comparisons of these characteristics by age (<70 years vs. >80 years) and sex are presented in Table 1. During the pandemic, 23.1% of those aged <70 years and 36.8% of those >80 years reported a reduction in light PA, while 36.3% of persons aged <70 years and 22.0% of those >80 years reported a reduction in higher-intensity PA (*P*≤0.001). Across sexes, 32.4% of women and 22.7% of men reported reductions in light PA (*P*≤0.05). There were no differences in higher-intensity PA reductions between women and men (*P*=0.97) (data not shown).

**Table 1:** Baseline characteristics of the participants by age and sex, N=624

	<70 years *n*= 347	>80 years *n*= 277	*P*-value^a^	Women *n*=413	Men *n*=211	*P*-value^a^
	n (%)	n (%)		n (%)	n (%)	
**Age, median (min-max)**	66.1 (65.9-68.4)	84.0 (80.9-99.2)	**<0.001**^b^	66.7 (65.9-99.2)	66.5 (65.9-96.1)	0.325^b^
**Sex**						
Women	225 (64.8)	188 (67.9)	0.427	-	-	
**Education**						
High school or less	112 (32.3)	146 (52.7)	**<0.001**	188 (45.5)	70 (33.2)	**0.003**
**Social network**						
Poor social support (< median)	155 (44.7)	157 (56.7)	**0.003**	194 (47.0)	118 (55.9)	**0.034**
Poor social connection (< median)	158 (45.5)	154 (55.6)	**0.012**	216 (52.3)	96 (45.5)	0.108
**Somatic diseases**						
Any cardiovascular disease	42 (12.1)	118 (42.6)	**<0.001**	88 (21.3)	72 (34.1)	**<0.001**
Any musculoskeletal disease	146 (42.1)	206 (74.4)	**<0.001**	237 (57.4)	115 (54.5)	0.492
**Mental diseases**						
Any neuropsychiatric disease	58 (16.7)	55 (19.9)	0.311	83 (20.1)	30 (14.2)	0.071
MMSE^c^ cut-off < 28	22 (6.3)	74 (26.7)	**<0.001**	59 (14.3)	37 (17.5)	0.287
MADRS^d^ cut-off > 6	23 (6.6)	29 (10.5)	0.085	40 (9.7)	12 (5.7)	0.087
**Physical functioning**						
Impaired mobility (< 0.8 m/seconds)	7 (2.0)	70 (25.3)	**<0.001**	62 (15.0)	15 (7.1)	**0.005**
Impaired balance (< 5 seconds)	27 (7.8)	159 (57.4)	**<0.001**	122 (29.5)	64 (30.3)	0.838
Impaired strength (≥ 17 seconds)	15 (4.3)	91 (32.9)	**<0.001**	72 (17.4)	34 (16.1)	0.678
**Lifestyle factors**						
Current smoker	35 (10.1)	9 (3.2)	**<0.001**	25 (6.1)	19 (9.0)	0.173
No/occasional or heavy alcohol consumption	115 (33.1)	132 (47.7)	**<0.001**	204 (49.4)	43 (20.4)	**<0.001**
Under- or overweight (BMI^e^ < 20 or ≥ 25 kg/m^2^)	189 (54.5)	163 (58.8)	0.273	224 (54.2)	128 (60.7)	0.126
**Personality**						
High/moderate neuroticism	200 (57.6)	159 (57.4)	0.953	254 (61.5)	105 (49.8)	**0.005**
Low extraversion	84 (24.2)	65 (23.5)	0.829	93 (22.5)	56 (26.5)	0.265
Low openness to experience	132 (38.0)	72 (26.0)	**0.001**	119 (28.8)	85 (40.3)	**0.004**
**Physical activity (PA)**^f^						
Insufficient PA	27 (7.8)	55 (20.4)	**<0.001**	52 (12.7)	30 (14.4)	0.562

Data are presented as the number of cases (n), the proportion (%), or the median with minimum to maximum range.^a^ Comparisons of characteristics between those aged <70 years and >80 years and women and men using Chi-Square tests and ^b^ Mann Whitney U tests. Abbreviations: ^c^ Mini-Mental State Examination, ^d^ The Montgomery-Åsberg Depression Rating Scale, ^e^ Body Mass Index. ^f^ Missing data for 8 persons.

Missing value analyses showed that those with missing data (*n*=301) were older, more likely to have any musculoskeletal disease or lower MMSE scores at baseline, as compared to the analytical sample (*n*=624). However, those in the analytical sample were more likely to report reductions in intense PA during the pandemic (*P*≤0.05). No differences were found for sex, educational levels, presence of any cardiovascular or neuropsychiatric diseases at baseline, or reduction in light PA during the pandemic between the analytical- and missing value samples (*P*≥0.05, data not shown).

There were significant interactions (*P*<0.05) between age (<70 vs >80 years) and having any musculoskeletal disease, and BMI (under- or overweight vs normal weight) with reductions in intense PA, and marginally significant interactions (*P*>0.05 and <0.1) between age (<70 vs >80 years) and depressive symptoms (MADRS >6 vs ≤6), and alcohol consumption (no/occasional or heavy vs light-moderate) with reductions in light PA. Moreover, there were significant interactions between sex and having any neuropsychiatric disease, and marginally significant interactions between sex and depressive symptoms (MADRS >6 vs ≤6), neuroticism (high/average vs low), and openness to experience (low vs high/average) with a reduction in light PA, as well as for any musculoskeletal disease, and neuroticism (high/average vs low) with reductions in intense PA (Supplementary Table 1). Therefore, further analyses were performed stratified by age and sex.

Table 2 shows the association between each pre-pandemic factor at baseline and reductions in light and intense PA during the COVID-19 pandemic by age. All models were adjusted for age, sex, and education. Among those aged <70 years, higher depressive symptoms scores were associated with a reduction in light-intensity PA (OR: 2.8, 95% CI: 1.1-6.7). Moreover, the presence of any musculoskeletal disease was related to an increased risk of reduction in higher-intensity PA (OR: 2.0, 95% CI: 1.3-3.1), while those who were current smokers were less likely to reduce their levels of higher-intensity PA (OR: 0.3, 95% CI: 0.1-0.8). A marginally significant association between high/moderate levels of neuroticism and an increased risk of reduction in higher-intensity PA (OR: 1.6, 95% CI: 1.0-2.5, *P*=0.052) was also detected.

**Table 2:** Adjusted odds ratios (OR)^a^ with 95% confidence intervals (95% CI) for the association between pre-pandemic factors at baseline and reduction in physical activity (PA) during the COVID-19 pandemic by age and intensity of PA, *N*=624

	Reduction in light PA						Reduction in intense PA					
	<70 years ***n***=347			>80 years ***n***=277			<70 years ***n***=347			>80 years ***n***=277		
	Reducers/non-reducers^b^	OR (95% CI)	*P*-value	Reducers/non-reducers^b^	OR (95% CI)	*P*-value	Reducers/non-reducers^b^	OR (95% CI)	*P*-value	Reducers/non-reducers^b^	OR (95% CI)	*P*-value
**Social network**												
Poor social support	37/118	1.1 (0.7-1.9)	0.625	56/101	0.9 (0.5-1.5)	0.663	58/97	1.1 (0.7-1.7)	0.743	41/116	**1.9 (1.0-3.6)**	**0.036**
Poor social connection	37/121	1.0 (0.6-1.7)	0.916	64/90	1.5 (0.9-2.6)	0.107	50/108	0.7 (0.4-1.0)	0.078	33/121	0.9 (0.5-1.7)	0.861
**Somatic diseases**												
Any cardiovascular disease	14/28	2.0 (1.0-4.1)	0.061	46/72	1.3 (0.8-2.2)	0.331	13/29	0.8 (0.4-1.6)	0.501	23/95	0.8 (0.5-1.6)	0.585
Any musculoskeletal disease	34/112	1.0 (0.6-1.7)	0.876	78/128	1.1 (0.6-2.0)	0.654	66/80	**2.0 (1.3-3.1)**	**0.003**	44/162	0.9 (0.5-1.8)	0.798
**Mental diseases**												
Any neuropsychiatric disease	14/44	1.0 (0.5-2.0)	0.893	20/35	0.9 (0.5-1.8)	0.860	21/37	1.0 (0.6-1.8)	0.987	15/40	1.3 (0.7-2.7)	0.412
MMSE^d^ <28	3/19	0.5 (0.2-1.9)	0.332	24/50	0.8 (0.4-1.4)	0.365	5/17	0.5 (0.2-1.4)	0.186	13/61	0.7 (0.3-1.4)	0.314
MADRS^e^ >6	10/13	**2.8 (1.1-6.7)**	**0.023**	10/19	0.8 (0.4-1.9)	0.686	9/14	1.1 (0.4-2.6)	0.864	6/23	1.0 (0.4-2.6)	0.990
**Physical functioning**												
Impaired mobility	2/5	1.2 (0.2-6.7)	0.799	26/44	0.9 (0.5-1.7)	0.758	1/6	0.2 (0.03-2.1)	0.198	11/59	0.7 (0.3-1.5)	0.337
Impaired balance	8/19	1.4 (0.6-3.5)	0.422	66/93	1.7 (1.0-2.8)	0.051	10/17	1.0 (0.4-2.2)	0.937	25/134	**0.5 (0.3-0.9)**	**0.016**
Impaired strength	4/11	1.3 (0.4-4.2)	0.671	35/56	1.1 (0.6-1.9)	0.773	5/10	0.8 (0.3-2.6)	0.765	15/76	0.7 (0.4-1.4)	0.319
**Lifestyle factors**												
Current smoker	8/27	1.0 (0.4-2.3)	0.999	0/9	-^c^		6/29	**0.3 (0.1-0.8)**	**0.011**	2/7	0.9 (0.2-4.8)	0.948
No/occasional or heavy alcohol consumption	34/81	1.6 (0.9-2.7)	0.094	46/86	0.7 (0.4-1.2)	0.219	38/77	0.8 (0.5-1.2)	0.257	25/107	0.7 (0.4-1.3)	0.310
Under- or overweight	49/140	1.5 (0.9-2.6)	0.112	64/99	1.3 (0.8-2.2)	0.273	76/113	1.4 (0.9-2.3)	0.108	31/132	0.6 (0.3-1.1)	0.097
**Personality**												
High/moderate neuroticism	47/153	1.0 (0.6-1.7)	0.860	63/96	1.2 (0.7-2.1)	0.411	81/119	1.6 (1.0-2.5)	0.052	40/119	1.8 (0.9-3.3)	0.076
Low extraversion	24/60	1.5 (0.9-2.7)	0.141	23/42	0.9 (0.5-1.7)	0.819	27/57	0.8 (0.5-1.3)	0.346	16/49	1.2 (0.6-2.3)	0.631
Low openness to experience	31/101	1.0 (0.6-1.8)	0.879	26/46	1.0 (0.6-1.9)	0.892	50/82	1.1 (0.7-1.7)	0.808	15/57	0.9 (0.4-1.8)	0.743

^a^ Controlled for age, sex and education in all analyses, ^b^ Number of reducers/non-reducers for each risk factor, ^c^ 0 persons in this category: reduced light PA and current smoker. Abbreviations: ^d^ Mini-Mental State Examination, ^e^ The Montgomery-Åsberg Depression Rating Scale.

For individuals aged >80 years, there was a marginally significant association between having impaired balance and an increased risk of reduction in light PA (OR: 1.7, 95% CI: 1.0-2.8, *P*=0.051). Also, pre-pandemic levels of poor social support were related with a reduction in higher-intensity PA (OR: 1.9, 95% CI: 1.0-3.6), while those having impaired balance were less likely to reduce their levels of higher-intensity PA (OR: 0.5, 95% CI: 0.3-0.9).

For women, there were no statistically significant associations with reductions in light PA, but high/moderate levels of neuroticism were associated with reductions in intense PA (OR: 2.0, 95% CI: 1.3-3.3). Moreover, those with impaired balance (OR: 0.5, 95% CI: 0.3-1.0) or current smokers (OR: 0.2, 95% CI: 0.06-0.7) were less likely to reduce their levels of intense PA.

For men, higher depressive burden was associated with reductions in light PA (OR: 3.4, 95% CI: 1.0-11.3), and having any musculoskeletal disease (OR: 2.3, 95% CI: 1.2-4.4) were associated with reductions in intense PA (Supplementary Table 2).

Table 3 and Supplementary Table 3 shows the fully adjusted models for the associations between pre-pandemic factors and reductions in light and intense PA during the pandemic by age and sex. All the results remained from the basic adjusted models except for the association between impaired balance and intense PA among women (OR: 0.6, 95% CI: 0.3-1.0, *P*=0.07), and higher depressive burden and reductions in light PA among men (OR: 2.7, 95% CI: 0.8-9.5, *P*=0.12), which were attenuated.

**Table 3:** Final multiadjusted odds ratios (OR) with 95% confidence intervals (95% CI) for the association between pre-pandemic factors at baseline and reduction in physical activity (PA) during the COVID-19 pandemic by age and intensity of PA, *N*=624^a^

	Reduction in light PA				Reduction in intense PA			
	<70 years ***n***=347		>80 years ***n***=277		<70 years ***n***=347		>80 years ***n***=277	
	OR (95% CI)	*P*-value	OR (95% CI)	*P*-value	OR (95% CI)	*P*-value	OR (95% CI)	*P*-value
**Social network**								
Poor social support	**-**		**-**		-		**2.2 (1.2-4.3)**	**0.016**
Poor social connection	**-**		**-**		0.7 (0.4-1.1)	0.143	**-**	
**Somatic diseases**								
Any cardiovascular disease	2.0 (0.9-4.1)	0.071	**-**		**-**		**-**	
Any musculoskeletal disease	**-**		**-**		**1.8 (1.1-2.9)**	**0.011**	**-**	
**Mental diseases**								
MADRS^b^ >6	**2.6 (1.1-6.4)**	**0.033**	**-**		**-**		**-**	
**Physical functioning**								
Impaired balance	**-**		1.7 (1.0-2.8)	0.051	**-**		**0.4 (0.2-0.8)**	**0.009**
**Lifestyle factors**								
Current smoker	**-**		**-**		**0.3 (0.1-0.8)**	**0.017**	**-**	
No/occasional or heavy alcohol consumption	1.5 (0.9-2.7)	0.121	**-**		**-**		**-**	
Under- or overweight	**-**		**-**		**-**		0.6 (0.3-1.1)	0.110
**Personality**								
High/moderate neuroticism	**-**		**-**		1.6 (1.0-2.6)	0.052	1.6 (0.9-3.1)	0.130

^a ^Final multiadjusted models from backward elimination procedure with a <0.1 p-value significance limit. Controlled for age, sex and education in all analyses. Abbreviations: ^b^ The Montgomery-Åsberg Depression Rating Scale

Sensitivity analyses were performed to investigate whether baseline PA levels influenced the main findings. When controlling for baseline PA, all the results remained for those <70 years of age. For individuals aged >80 years, all results remained except for the association with reduction in light PA where the association with impaired balance was attenuated (1.6, 95% CI: 0.9-2.7, *P*=0.10).

## Discussion

In this urban population of older adults aged 65-99 years old, we found an association between pre-pandemic levels of higher depressive symptom burden (<70 years old), and impaired balance (>80 years old) with reductions in light-intensity PA during the first wave of the pandemic. Moreover, the pre-pandemic presence of any musculoskeletal disease (<70 years and men), higher levels of neuroticism, being a non-smoker (<70 years old and women), poor levels of social support, or having a good balance (>80 years old) were associated with reductions in higher-intensity PA during the pandemic.

To the best of our knowledge, this is the first study investigating pre-pandemic factors associated with reductions of PA levels in a population-based sample of older adults. However, our findings corroborate and extend results from recent studies showing that pre-pandemic levels of mental health, frailty-related symptoms, and social contacts were associated with changes in PA levels during the COVID-19 outbreak among frail older adults and individuals at risk of developing dementia [6, 10]. Furthermore, we found that differential factors were associated with reduction in light versus intense PA. Our findings are supported by previous research showing different correlates for light and intense PA among older adults [14, 36, 37].

We found that higher pre-pandemic levels of depressive symptoms were associated with a reduction in light-intensity PA in those aged <70 years. Our results are in accordance with previous research showing depression to be inversely associated with PA [12], and with the results from Perez et al., who found higher depressive symptoms scores to be related to reductions or maintenance of insufficient PA during the COVID-19 pandemic [10]. The association between depressive symptom burden and reductions in light-intensity PA was only detected in the younger age group (<70 years) and may be explained by older individuals’ preference for positive over negative stimuli in cognitive processing [38]. This might make younger-old individuals more prone to change their participation in healthy behaviors, such as PA, when being exposed to higher levels of depressive symptoms, as compared to the oldest-old individuals.

Our results that impaired balance was marginally associated with a reduction in light-intensity PA among the oldest-old during the pandemic is consistent with previous literature, showing that deficits in balance seem to be a strong predictor of activity limitations in older adults [39]. By contrast, we found that having a good balance was associated with a reduction in higher-intensity PA in the same age group. Considering that the oldest-old individuals with poor balance are less likely to engage in higher-intensity PA, this finding is not unexpected. Previous research has shown that persons with musculoskeletal diseases in general spend most of their time in sedentary activities [40]. The present study further showed that adults younger than 70 years and men who had a musculoskeletal disease were more likely to reduce their participation in higher-intensity PA. Considering that PA is a key component in the treatment of musculoskeletal disease [41], this reduction may have particularly detrimental effects.

Previous studies on the association between social support and PA in older adults have produced inconsistent results [42]. However, our finding that poor pre-pandemic levels of social support were associated with a reduction in higher-intensity PA during the pandemic among those aged >80 years, is partly in line with a previous study from our group. This study suggests that higher levels of social support correlate positively with more time spent in light PA, especially among the oldest-old individuals [37]. Our results are also partly in line with a Finnish study showing that those who lived alone were more susceptible to reduce their levels of PA and physical functioning during the first outbreak [6], and a Spanish study showing that social contacts outside the family were related to an improvement or maintenance of sufficient PA during the first wave of the pandemic [10].

Surprisingly, we found that being a non-smoker was associated with a reduction in higher-intensity PA during the pandemic in those aged <70 years and among women. Previous research indicates that smoking is unrelated to PA [43]. It has however been suggested that nicotine may induce an increased risk seeking behavior [44], which could provide one possible explanation to our finding of smokers being more likely to perform higher-intensity PA during the pandemic, e.g., being more likely to visit a gym. However, since the number of smokers was relatively low, this finding should be interpretated with caution and verified in future studies. Moreover, our finding that those <70 years and women with moderate/high neurotic personality-traits had a tendency to reduce their levels of high-intensity PA during the pandemic is in line with studies showing that persons with higher levels of neuroticism are more risk averse than those with lower levels of neuroticism [45].

Major strengths of this study are the large population-based sample of older individuals with a wide age-range (65-99 years), which enabled stratified analyses. Furthermore, the longitudinal study design made it possible to study temporality and minimized the possibility of recall bias. Some limitations also need to be addressed. First, this work used a telephone interview to assess changes in PA levels, and respondent bias might therefore not be fully discarded. Second, Kungsholmen is a relatively affluent area in Stockholm, Sweden, and those with missing data were older and less healthy, as compared to the analytical sample. Nevertheless, these potential limitations may, if anything, have led to an underestimation of the associations of pre-pandemic levels of physical, mental, social and lifestyle factors with reductions in PA. Third, the outcome measure was collected during a specific time of the year (May-September). Since individuals may be more active during the spring and summer [46], this may have led to an underestimation of the reduction in PA since, when asked about differences with pre-pandemic PA levels, participants may have taken the preceding fall or winter period as a reference, when PA levels might already have been lower. Fourth, even though we applied a backward elimination procedure in our multiple regression models, whereby we only included those risk factors with P-values <0.1 in univariate associations with PA reductions, we cannot eliminate the possibility of type 2 error. Fifth, no further assessments of the exposures were available between the baseline assessment (years 2016-2019) and SNAC-K COVID19 Study (May-September 2020), which limited our possibility to explore potential changes in the exposure variables until the beginning of the pandemic. Finally, the dichotomization of the exposures and outcome may have reduced sensitivity due to loss of information. However, this eased the overall interpretability and increased the power of the study.

## Conclusions

We found that pre-pandemic levels of depressive symptoms and impaired balance were related to a reduction in light-intensity PA, while the presence of any musculoskeletal disease, higher levels of neuroticism, and poor levels of social support were associated with reductions in higher-intensity PA during the first wave of the pandemic. More population-based studies in different settings and countries are needed to further confirm these results. Taken together, for future pandemics or waves of COVID-19, development of strategies and specific attention from society and health care is warranted for older individuals with psychiatric- or physical illness/dysfunction, as well as those with poor social support to counteract reductions in physical activities.

## Supplementary Information

**Additional file 1: Supplementary Table 1.** Adjusted odds ratios (OR) with 95% confidence intervals (95% CI) for the association between pre-pandemic factors at baseline and reduction in physical activity (PA) during the Covid-19 pandemic by intensity of PA, *N*=624^a^.

**Additional file 2: Supplementary Table 2.** Adjusted odds ratios (OR)^a^ with 95% confidence intervals (95% CI) for the association between pre-pandemic factors at baseline and reduction in physical activity (PA) during the COVID-19 pandemic by sex and intensity of PA, *N*=624.

**Additional file 3: Supplementary Table 3.** Final multiadjusted odds ratios (OR) with 95% confidence intervals (95% CI) for the association between pre-pandemic factors at baseline and reduction in physical activity (PA) during the COVID-19 pandemic by sex and intensity of PA, *N*=624^a^.

## Data Availability

Data are from the SNAC-K (https://www.snac-k.se/) project, a population-based studies on aging and dementia. Access to the original data is available to the research community upon approval by the SNAC-K data management and maintenance committee. Application for accessing the data can be submitted to Maria Wahlberg (Maria.Wahlberg@ki.se) at the Aging Research Center, Karolinska Institutet.
