# Supplementary material for: Factors associated with physical activity reduction in Swedish older adults during the first COVID-19 outbreak: a longitudinal population-based study
Source: Eur Rev Aging Phys Act. 2022 Apr 1;19:9. doi: 10.1186/s11556-022-00287-z (PMC8972725; doi:10.1186/s11556-022-00287-z)
Supplement: Supplementary file 1 — Additional file 1: Supplementary Table 1. Adjusted odds ratios (OR) with 95% confidence intervals (95% CI) for the association between pre-pandemic factors at baseline and reduction in physical activity (PA) during the Covid-19 pandemic by intensity of PA, N=624a. [file 11556_2022_287_MOESM1_ESM.docx]

**Supplementary Table 1.** Adjusted odds ratios (OR) with 95% confidence intervals (95% CI) for the association between pre-pandemic factors at baseline and reduction in physical activity (PA) during the Covid-19 pandemic by intensity of PA, N=624^a^

|  | **Reduction in light PA** | | |  |  | **Reduction in intense PA** | | |  |
| --- | --- | --- | --- | --- | --- | --- | --- | --- | --- |
|  | OR (95% CI) | P-value | Age interaction P-value^b^ | Sex interaction P-value^c^ |  | OR (95% CI) | P-value | Age interaction P-value^b^ | Sex interaction P-value^c^ |
| **Social network** |  |  |  |  |  |  |  |  |  |
| Poor social support | 1.0 (0.7-1.4) | 0.985 | 0.506 | 0.879 |  | 1.3 (0.9-1.9) | 0.154 | 0.217 | 0.849 |
| Poor social connection | 1.2 (0.9-1.8) | 0.233 | 0.296 | 0.245 |  | 0.7 (0.5-1.1) | 0.111 | 0.487 | 0.583 |
| **Somatic diseases** |  |  |  |  |  |  |  |  |  |
| Any cardiovascular disease | 1.5 (1.0-2.3) | 0.060 | 0.305 | 0.593 |  | 0.8 (0.5-1.3) | 0.407 | 0.987 | 0.260 |
| Any musculoskeletal disease | 1.1 (0.8-1.6) | 0.627 | 0.803 | 0.274 |  | **1.6 (1.1-2.3)** | **0.016** | **0.043** | *0.091* |
| **Mental diseases** |  |  |  |  |  |  |  |  |  |
| Any neuropsychiatric disease | 1.0 (0.6-1.6) | 0.994 | 0.836 | **0.028** |  | 1.2 (0.7-1.8) | 0.499 | 0.427 | 0.217 |
| MMSE^d^ <28 | 0.7 (0.4-1.2) | 0.198 | 0.610 | 0.993 |  | 0.6 (0.4-1.1) | 0.115 | 0.653 | 0.239 |
| MADRS^e^ >6 | 1.4 (0.8-2.5) | 0.287 | *0.064* | *0.095* |  | 1.0 (0.5-1.9) | 0.958 | 0.777 | 0.569 |
| **Physical functioning** |  |  |  |  |  |  |  |  |  |
| Impaired mobility | 0.9 (0.5-1.6) | 0.742 | 0.779 | 0.415 |  | 0.6 (0.3-1.1) | 0.096 | 0.485 | 0.335 |
| Impaired balance | **1.6 (1.0-2.5)** | **0.033** | 0.810 | 0.856 |  | **0.6 (0.4-1.0)** | **0.043** | 0.107 | 0.341 |
| Impaired strength | 1.1 (0.7-1.8) | 0.760 | 0.842 | 0.958 |  | 0.7 (0.4-1.2) | 0.225 | 0.580 | 0.429 |
| **Lifestyle factors** |  |  |  |  |  |  |  |  |  |
| Current smoker | 0.6 (0.3-1.4) | 0.266 | - | 0.821 |  | **0.4 (0.2-0.8)** | **0.015** | 0.175 | 0.184 |
| No/occasional or heavy  alcohol consumption | 1.0 (0.7-1.5) | 0.816 | *0.057* | 0.139 |  | 0.7 (0.5-1.1) | 0.135 | 0.785 | 0.921 |
| Under- or overweight | 1.4 (1.0-2.0) | 0.056 | 0.722 | 0.409 |  | 1.1 (0.7-1.5) | 0.737 | **0.043** | 0.236 |
| **Personality** |  |  |  |  |  |  |  |  |  |
| High/average neuroticism | 1.1 (0.8-1.6) | 0.507 | 0.618 | *0.074* |  | **1.6 (1.1-2.3)** | **0.012** | 0.987 | *0.094* |
| Low extraversion | 1.2 (0.8-1.8) | 0.368 | 0.227 | 0.951 |  | 0.9 (0.6-1.4) | 0.645 | 0.296 | 0.926 |
| Low openness to experience | 1.0 (0.7-1.5) | 0.860 | 0.949 | *0.062* |  | 1.0 (0.7-1.4) | 0.920 | 0.626 | 0.561 |

^a^ Controlled for age, sex and education in all analyses. Interaction effects between each exposure variable with ^b^age and ^c^sex. Interactions with P-values ≤0.05 are marked in bold and P-values <0.1 in italics. Abbreviations: ^d^ Mini-Mental State Examination, ^e^ The Montgomery-Åsberg Depression Rating Scale.
