# Supplementary material for: Factors associated with physical activity reduction in Swedish older adults during the first COVID-19 outbreak: a longitudinal population-based study
Source: Eur Rev Aging Phys Act. 2022 Apr 1;19:9. doi: 10.1186/s11556-022-00287-z (PMC8972725; doi:10.1186/s11556-022-00287-z)
Supplement: Supplementary file 2 — Additional file 2: Supplementary Table 2. Adjusted odds ratios (OR)a with 95% confidence intervals (95% CI) for the association between pre-pandemic factors at baseline and reduction in physical activity (PA) during the COVID-19 pandemic by sex and intensity of PA, N=624. [file 11556_2022_287_MOESM2_ESM.docx]

**Supplementary Table 2.** Adjusted odds ratios (OR)^a^ with 95% confidence intervals (95% CI) for the association between pre-pandemic factors at baseline and reduction in physical activity (PA) during the COVID-19 pandemic by sex and intensity of PA, N=624.

|  | **Reduction in light PA** | | | | | |  | **Reduction in intense PA** | | | | | |
| --- | --- | --- | --- | --- | --- | --- | --- | --- | --- | --- | --- | --- | --- |
|  | **Women**  **n=413** | | | **Men**  **n=211** | | |  | **Women**  **n=413** | | | **Men**  **n=211** | | |
|  | Reducers/  non-reducers^b^ | OR (95% CI) | P-value | Reducers/  non-reducers^b^ | OR (95% CI) | P-value |  | Reducers/  non-reducers^b^ | OR (95% CI) | P-value | Reducers/  non-reducers^b^ | OR (95% CI) | P-value |
| **Social network** |  |  |  |  |  |  |  |  |  |  |  |  |  |
| Poor social support | 66/128 | 1.0 (0.7-1.5) | 0.974 | 27/91 | 1.0 (0.5-1.9) | 0.967 |  | 62/132 | 1.4 (0.9-2.1) | 0.167 | 37/81 | 1.2 (0.7-2.2) | 0.565 |
| Poor social connection | 80/136 | 1.4 (0.9-2.2) | 0.114 | 21/75 | 0.9 (0.5-1.7) | 0.767 |  | 59/157 | 0.8 (0.5-1.3) | 0.368 | 24/72 | 0.6 (0.4-1.2) | 0.159 |
| **Somatic diseases** |  |  |  |  |  |  |  |  |  |  |  |  |  |
| Any cardiovascular disease | 37/51 | 1.3 (0.8-2.2) | 0.292 | 23/49 | 1.9 (0.9-4.0) | 0.075 |  | 17/71 | 0.7 (0.4-1.3) | 0.242 | 19/53 | 1.0 (0.5-2.0) | 0.991 |
| Any musculoskeletal disease | 81/156 | 0.9 (0.6-1.5) | 0.747 | 31/84 | 1.6 (0.8-3.1) | 0.198 |  | 69/168 | 1.3 (0.8-2.1) | 0.246 | 41/74 | **2.3 (1.2-4.4)** | **0.011** |
| **Mental diseases** |  |  |  |  |  |  |  |  |  |  |  |  |  |
| Any neuropsychiatric disease | 23/60 | 0.7 (0.4-1.3) | 0.273 | 11/19 | 2.3 (1.0-5.2) | 0.055 |  | 29/54 | 1.4 (0.8-2.4) | 0.204 | 7/23 | 0.7 (0.3-1.8) | 0.455 |
| MMSE^c^ <28 | 19/40 | 0.7 (0.4-1.3) | 0.241 | 8/29 | 0.8 (0.3-1.9) | 0.552 |  | 9/50 | 0.5 (0.2-1.1) | 0.078 | 9/28 | 0.9 (0.4-2.0) | 0.733 |
| MADRS^d^ >6 | 14/26 | 1.0 (0.5-2.1) | 0.909 | 6/6 | **3.4 (1.0-11.3)** | **0.044** |  | 11/29 | 0.9 (0.4-1.9) | 0.812 | 4/8 | 1.4 (0.4-4.9) | 0.617 |
| **Physical functioning** |  |  |  |  |  |  |  |  |  |  |  |  |  |
| Impaired mobility | 25/37 | 1.0 (0.5-1.9) | 0.997 | 3/12 | 0.6 (0.1-2.3) | 0.420 |  | 11/51 | 0.7 (0.3-1.5) | 0.362 | 1/14 | 0.2 (0.02-1.5) | 0.118 |
| Impaired balance | 53/69 | 1.5 (0.9-2.5) | 0.134 | 21/43 | 2.0 (0.9-4.7) | 0.096 |  | 21/101 | **0.5 (0.3-1.0)** | **0.050** | 14/50 | 0.7 (0.3-1.6) | 0.420 |
| Impaired strength | 29/43 | 1.0 (0.6-1.9) | 0.923 | 10/24 | 1.2 (0.5-3.0) | 0.678 |  | 15/57 | 0.9 (0.5-1.8) | 0.786 | 5/29 | 0.4 (0.1-1.2) | 0.103 |
| **Lifestyle factors** |  |  |  |  |  |  |  |  |  |  |  |  |  |
| Current smoker | 5/20 | 0.6 (0.2-1.7) | 0.320 | 3/16 | 0.7 (0.2-2.5) | 0.574 |  | 3/22 | **0.2 (0.06-0.7)** | **0.012** | 5/14 | 0.7 (0.2-2.1) | 0.526 |
| No/occasional or heavy  alcohol consumption | 66/138 | 0.9 (0.6-1.4) | 0.583 | 14/29 | 1.8 (0.9-3.8) | 0.124 |  | 53/151 | 0.8 (0.5-1.2) | 0.240 | 10/33 | 0.7 (0.3-1.5) | 0.358 |
| Under- or overweight | 79/145 | 1.3 (0.8-2.0) | 0.229 | 34/94 | 1.8 (0.9-3.6) | 0.099 |  | 65/159 | 0.9 (0.6-1.4) | 0.684 | 42/86 | 1.5 (0.8-2.7) | 0.242 |
| **Personality** |  |  |  |  |  |  |  |  |  |  |  |  |  |
| High/moderate neuroticism | 90/164 | 1.4 (0.9-2.2) | 0.121 | 20/85 | 0.7 (0.3-1.3) | 0.223 |  | 89/165 | **2.0 (1.3-3.3)** | **0.003** | 32/73 | 1.1 (0.6-2.0) | 0.829 |
| Low extraversion | 33/60 | 1.2 (0.7-2.0) | 0.460 | 14/42 | 1.2 (0.6-2.5) | 0.611 |  | 27/66 | 0.9 (0.5-1.5) | 0.729 | 16/40 | 0.9 (0.5-1.8) | 0.764 |
| Low openness to experience | 42/77 | 1.3 (0.8-2.1) | 0.228 | 15/70 | 0.6 (0.3-1.2) | 0.169 |  | 40/79 | 1.1 (0.7-1.7) | 0.829 | 25/60 | 0.9 (0.5-1.6) | 0.619 |

^a^ Controlled for age, sex and education in all analyses, ^b^ Number of reducers/non-reducers for each risk factor. Abbreviations: ^c^ Mini-Mental State Examination, ^d^ The Montgomery-Åsberg Depression Rating Scale.
