# Supplementary material for: Factors associated with physical activity reduction in Swedish older adults during the first COVID-19 outbreak: a longitudinal population-based study
Source: Eur Rev Aging Phys Act. 2022 Apr 1;19:9. doi: 10.1186/s11556-022-00287-z (PMC8972725; doi:10.1186/s11556-022-00287-z)
Supplement: Supplementary file 3 — Additional file 3: Supplementary Table 3. Final multiadjusted odds ratios (OR) with 95% confidence intervals (95% CI) for the association between pre-pandemic factors at baseline and reduction in physical activity (PA) during the COVID-19 pandemic by sex and intensity of PA, N=624a. [file 11556_2022_287_MOESM3_ESM.docx]

**Supplementary Table 3.** Final multiadjusted odds ratios (OR) with 95% confidence intervals (95% CI) for the association between pre-pandemic factors at baseline and reduction in physical activity (PA) during the COVID-19 pandemic by sex and intensity of PA, N=624^a^

|  | **Reduction in light PA** | | | |  | **Reduction in intense PA** | | | | |
| --- | --- | --- | --- | --- | --- | --- | --- | --- | --- | --- |
|  | **Women**  **n=413** | | **Men**  **n=211** | |  | **Women**  **n=413** |  | **Men**  **n=211** |  |  |
|  | OR (95% CI) | P-value | OR (95% CI) | P-value |  | OR (95% CI) | P-value | OR (95% CI) | P-value |  |
| **Somatic diseases** |  |  |  |  |  |  |  |  |  |  |
| Any cardiovascular disease | - |  | 1.7 (0.8-3.6) | 0.172 |  | **-** |  | **-** |  |  |
| Any musculoskeletal disease | - |  | **-** |  |  | **-** |  | **2.3 (1.2-4.4)** | **0.011** |  |
| **Mental diseases** |  |  |  |  |  |  |  |  |  |  |
| Any neuropsychiatric disease | **-** |  | 1.9 (0.8-4.4) | 0.167 |  | **-** |  | **-** |  |  |
| MMSE^b^ <28 | **-** |  | **-** |  |  | 0.5 (0.2-1.2) | 0.132 | **-** |  |  |
| MADRS^c^ >6 | **-** |  | 2.7 (0.8-9.5) | 0.115 |  | **-** |  | **-** |  |  |
| **Physical functioning** |  |  |  |  |  |  |  |  |  |  |
| Impaired balance | **-** |  | 1.4 (0.6-3.5) | 0.420 |  | 0.6 (0.3-1.0) | 0.065 | **-** |  |  |
| **Lifestyle factors** |  |  |  |  |  |  |  |  |  |  |
| Current smoker | **-** |  | **-** |  |  | **0.2 (0.06-0.7)** | **0.013** | **-** |  |  |
| Under- or overweight | **-** |  | 1.7 (0.8-3.5) | 0.158 |  | **-** |  | **-** |  |  |
| **Personality** |  |  |  |  |  |  |  |  |  |  |
| High/moderate neuroticism | **-** |  | **-** |  |  | **2.2 (1.3-3.5)** | **0.002** |  |  |  |

^a^ Final multiadjusted models from backward elimination procedure with a <0.1 p-value significance limit. Controlled for age, sex and education in all analyses. Abbreviations: ^b^ Mini-Mental State Examination, ^c^ The Montgomery-Åsberg Depression Rating Scale.
